# Design of Well‐Defined Meso‐ or Macroporous Carbon Nitride with an Amorphous Framework via Perovskite Fluoride Templating

**DOI:** 10.1002/smll.74242

**Published:** 2026-06-19

**Authors:** Kazuya Kozumi, Yusuke Asakura, Yusuke Yamauchi

**Affiliations:** ^1^ Department of Materials Process Engineering Graduate School of Engineering Nagoya University Nagoya Aichi Japan; ^2^ Australian Institute for Bioengineering and Nanotechnology (AIBN) The University of Queensland Brisbane Queensland Australia

## Abstract

Here, we report a synthetic strategy for meso‐ or macroporous carbon nitride (C_3_N_4_) using perovskite‐type metal fluorides (K*M*F_3_, *M* = Co, Ni) as hard templates. The synthesis involves a two‐step thermal treatment. An initial low‐temperature polycondensation of melamine generates polymeric intermediates that stabilize the template/precursor composites, thereby suppressing aggregation and/or sintering of K*M*F_3_ during the subsequent high‐temperature treatment. The subsequent high‐temperature treatment then induces further condensation into C_3_N_4_ while retaining the template‐defined structures. After removal of the templates, meso‐ or macroporous C_3_N_4_ are successfully obtained, demonstrating that K*M*F_3_ can serve as effective templates even under high‐temperature polycondensation conditions through appropriate reaction stage control. As a representative demonstration of functionality, electrochemical glucose sensing was used to evaluate the porous materials. The mesoporous KNiF_3_‐templated sample (Mel‐KNiF_3_) shows higher sensitivity and a lower detection limit than bulk C_3_N_4_ and the macroporous KCoF_3_‐templated sample (Mel‐KCoF_3_), suggesting an important role of pore architecture in determining the accessibility of active sites. This study establishes a practical route to porous carbon nitride under thermal conditions and provides a simple strategy for structural control in high‐temperature polymeric systems.

## 1 Introduction

Carbon nitride (C_3_N_4_), a polymeric semiconductor composed of earth‐abundant carbon and nitrogen, has garnered significant attention as a promising functional material. It features a conjugated framework built from triazine or heptazine units, possessing a bandgap of approximately 2.7 eV together with exceptional chemical and thermal stability [1, 2]. These intrinsic properties make C_3_N_4_ highly attractive for various applications, including photocatalysis for reactions such as CO_2_ reduction [3, 4], and H_2_O_2_ production [5, 6], electrocatalysis for the oxygen reduction reaction (ORR) [7, 8], and sensing applications [9, 10, 11]. C_3_N_4_ is generally synthesized via the solid‐state thermal polymerization of low‐cost precursors such as melamine, urea, and cyanamide. By varying the precursor chemistry and polymerization conditions, the structural characteristics of C_3_N_4_ can be tuned, enabling control over its crystallinity from graphitic layered structures to amorphous polymeric networks [12]. In addition, heteroatom doping, such as with sulfur or metal species [13], has been widely explored to further enhance its activity. Moreover, morphological engineering, including the construction of porous structures [14], nanosheets [15], and nanoparticles [16], has been extensively investigated to improve reactant diffusion and charge transport, because conventional thermal polymerization typically produces bulk C_3_N_4_ with low specific surface areas and limited mass transport, thereby hindering efficient interactions between the material surface and reactant species. Therefore, both atomic‐level structural control and morphological engineering are essential for maximizing the functional performance of C_3_N_4_.

One important strategy in morphological engineering is the introduction of pores into materials. In porous materials, the function of pores strongly depends on their size. Macropores (>50 nm) primarily facilitate molecular diffusion but contribute little to the overall surface area. In contrast, micropores (<2 nm) provide large surface areas but often restrict diffusion due to their small dimensions. Mesopores (2–50 nm) increase surface area while maintaining efficient molecular transport and are therefore advantageous for applications involving interactions between the material surface and reactant species [17, 18, 19, 20]. Because soft‐templating methods cannot be readily applied to the synthesis of porous carbon nitrides, owing to the low thermal stability of organic templates under high‐temperature polycondensation conditions, hard‐templating approaches using silica (SiO_2_) templates have been widely employed [21, 22]. However, removal of silica templates typically requires harsh chemical treatment with HF or highly concentrated alkaline solutions. In particular, the use of HF raises serious safety concerns due to its extreme toxicity and corrosiveness. Consequently, the development of safer and more practical synthetic strategies for porous carbon nitride remains highly desirable.

Recently, we proposed that perovskite‐type fluorides (K*M*F_3_, *M* = Fe, Co, Ni, etc.) can serve as a new class of templates owing to their unique physicochemical properties. K*M*F_3_ nanoparticles can be synthesized with tunable particle sizes ranging from several tens to several hundreds of nanometers and readily dissolve in aqueous solution. These characteristics enable their facile removal by water treatment at room temperature. In our previous work, we demonstrated that K*M*F_3_ templates are effective for generating meso‐ or macroporous organic polymers via low‐temperature solid‐state polymerizations through Schiff‐base reactions [23]. However, the applicability of K*M*F_3_ templates to high‐temperature polycondensation systems has not yet been explored. In particular, their structural stability and templating capability under high‐temperature conditions involving precursor decomposition and polycondensation remain unclear.

**SCHEME 1:**
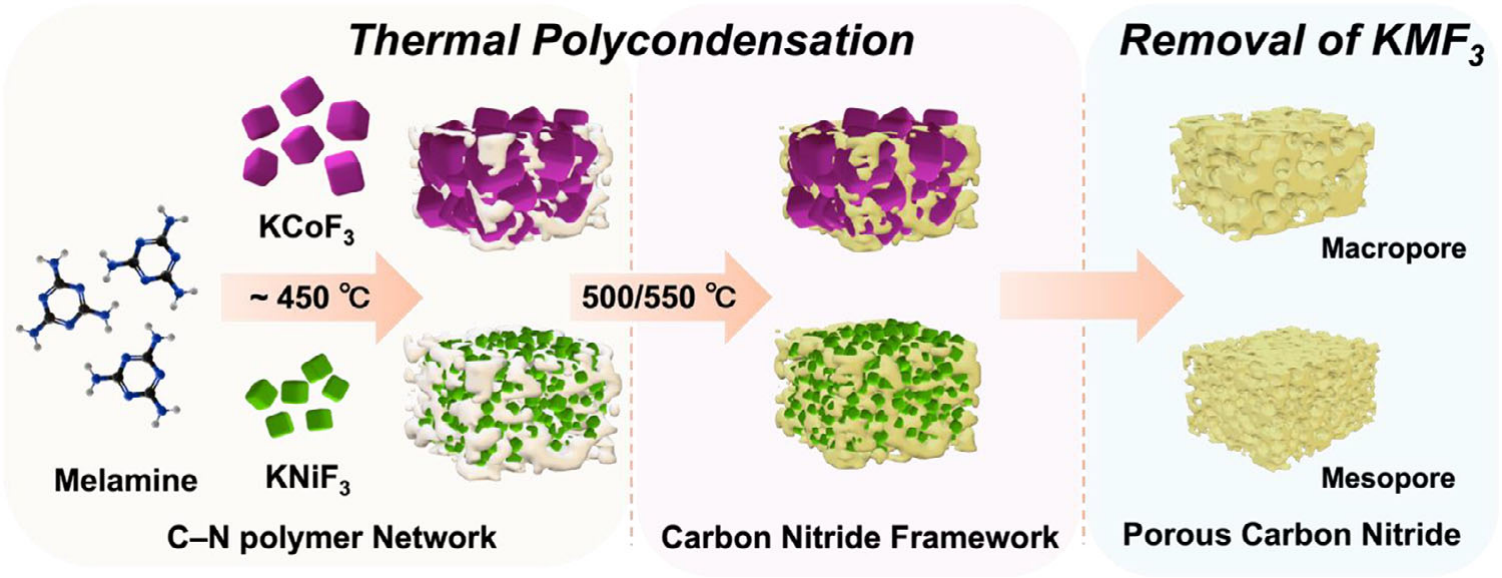
Construction of porous carbon nitride via thermal polycondensation of melamine using K*M*F_3_ templates.

In this study, we propose a strategy for the synthesis of meso‐ or macroporous C_3_N_4_ with an amorphous framework from melamine using K*M*F_3_ nanoparticles as templates. A key feature of this strategy is the introduction of an initial low‐temperature stage to promote the polymerization of melamine. At this stage, polymerization proceeds within the interparticle spaces of K*M*F_3_ nanoparticles, generating a C─N polymer network that surrounds the templates. The resulting framework stabilizes the spatial arrangement of the K*M*F_3_ nanoparticles during the subsequent high‐temperature treatment. After removal of the K*M*F_3_ templates, the template‐defined pore structures are faithfully replicated, yielding meso‐ or macroporous amorphous C_3_N_4_. In contrast, direct high‐temperature treatment leads to aggregation of the K*M*F_3_ templates, preventing the formation of well‐defined porous structures. These results indicate that the melamine‐derived polymer network formed at low temperature suppresses template aggregation during the high‐temperature polycondensation process, thereby enabling successful templating. The obtained C_3_N_4_ possesses an amorphous carbon nitride network with residual surface functional groups (─C≡N), which may impart additional unique surface properties. To examine the functional characteristics of the porous amorphous C_3_N_4_, electrochemical glucose sensing, known to be sensitive to the surface structure, was investigated. The porous samples exhibit significantly enhanced sensitivity compared with non‐porous counterparts. These results demonstrate that the K*M*F_3_‐templating strategy provides an effective route to fabricate porous carbon nitride structures through high‐temperature polymerization processes, enabling the creation of functional materials with unique properties (Scheme 1).

## 2 Results and Discussion

For the synthesis of meso‐ or macroporous materials, melamine and K*M*F_3_ (*M* = Co, Ni) were used as the precursor and templates, respectively. Polycondensation in the presence of an excess amount of K*M*F_3_ templates was conducted via a two‐stage process. In the first stage, a mixture of melamine and K*M*F_3_ was heated to 450°C, followed by further heating to 500°C or 550°C in the second stage. The obtained materials were denoted as Mel‐K*M*F_3_‐450‐500 or Mel‐K*M*F_3_‐450‐550, where *M* represents Co or Ni. For comparison, mixtures of melamine and K*M*F_3_ templates were directly heated at 450°C, 500°C, or 550°C and denoted as Mel‐K*M*F_3_‐*XXX*, where *XXX* indicates the heating temperature. Furthermore, non‐porous materials were synthesized at 500°C or 550°C without templates and are referred to as C_3_N_4_‐500 and C_3_N_4_‐550.

Scanning transmission electron microscopy (STEM) images of the KCoF_3_ and KNiF_3_ templates (Figure S1a,b) show that the particle sizes are approximately 50–200 nm and 10–30 nm, respectively. Both templates possess perovskite‐type crystal structures, as confirmed by X‐ray diffraction (XRD) patterns (Figure S2). Thermogravimetric (TG) analysis was first conducted to evaluate the thermal stability of K*M*F_3_. TG analysis and XRD measurements reveal negligible weight loss and no detectable phase transformation up to 600°C (Figures S3 and S4), confirming that K*M*F_3_ retains its crystalline structure within the temperature range required for C_3_N_4_ synthesis.

**FIGURE 1:**
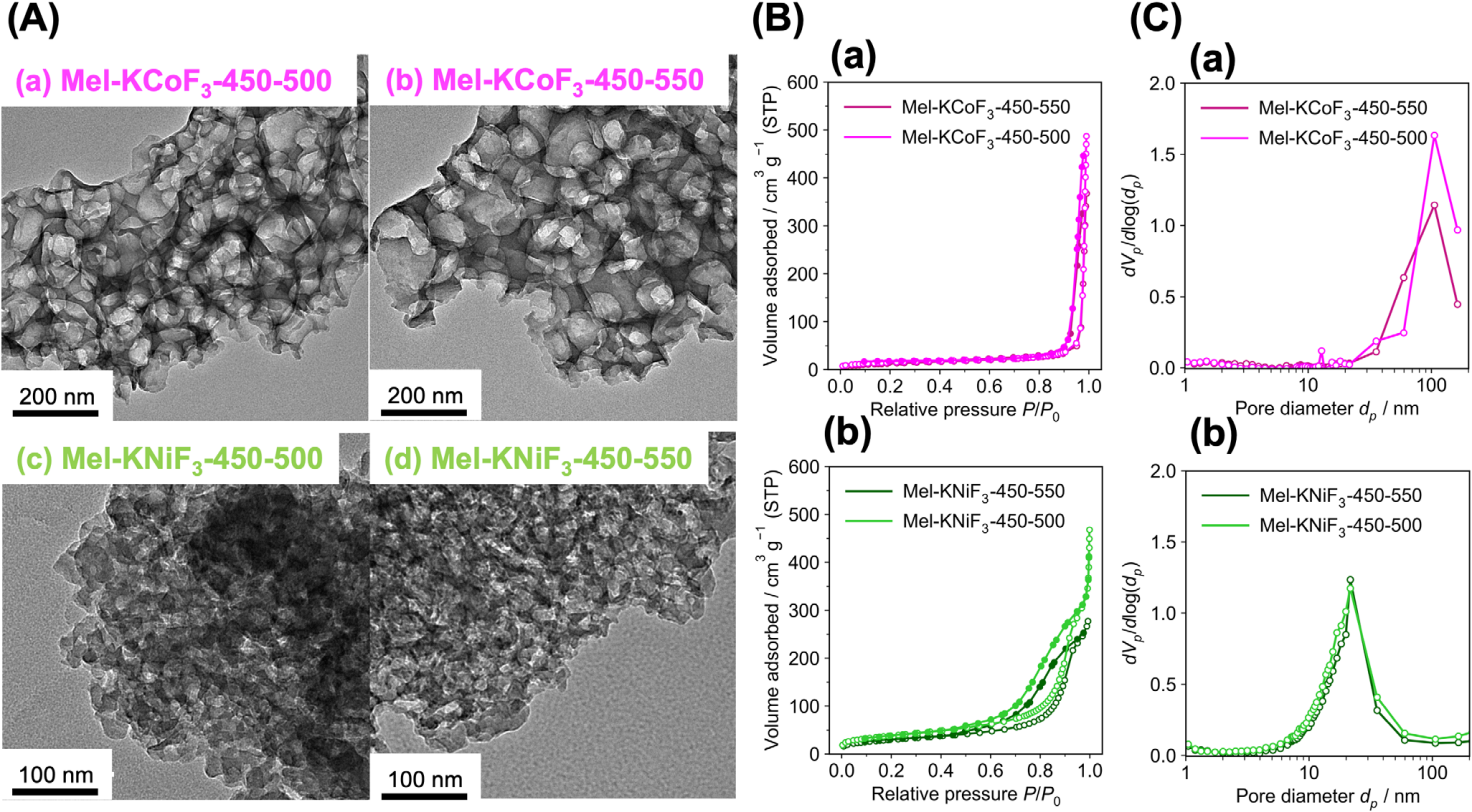
(A) TEM images of (a) Mel‐KCoF_3_‐450‐500, (b) Mel‐KCoF_3_‐450‐550, (c) Mel‐KNiF_3_‐450‐500, and (d) Mel‐KNiF_3_‐450‐550. (B) N_2_ adsorption–desorption isotherms and (C) BJH pore size distributions of each sample.

The morphology and porosity of the synthesized C_3_N_4_ samples prepared using K*M*F_3_ templates were characterized by transmission electron microscopy (TEM) and N_2_ adsorption–desorption measurements. Figure 1A–C shows the TEM images, adsorption–desorption isotherms, and Barrett‐Joyner‐Halenda (BJH) pore size distributions of the samples prepared by stepwise calcination using K*M*F_3_ (*M* = Co or Ni) as templates. The KCoF_3_‐templated samples (Mel‐KCoF_3_‐450‐500 and Mel‐KCoF_3_‐450‐550) exhibit well‐defined porous structures with pore sizes in the range of 50–200 nm (Figure 1A‐a,b). Similarly, the KNiF_3_‐templated samples (Mel‐KNiF_3_‐450‐500 and Mel‐KNiF_3_‐450‐550) show well‐defined porous structures with pore sizes of approximately 10–30 nm (Figure 1A‐c,d). These porous structures are closely correlated with the particle sizes of the corresponding templates, KCoF_3_ and KNiF_3_.

The N_2_ adsorption–desorption isotherms and BJH pore size distributions of the prepared samples are shown in Figure 1B,C. For Mel‐KCoF_3_‐450‐500 and Mel‐KCoF_3_‐450‐550, the isotherms exhibit a pronounced adsorption uptake at high relative pressures (*P*/*P*_0_ = 0.9–1.0), accompanied by a small hysteresis loop. Consistently, the BJH pore size distributions show broad features centered at approximately 100 nm. Although BJH analysis is less quantitative in this macropore range, the obtained feature in good agreement with the particle size of the KCoF_3_ templates, suggesting the formation of macroporous structures (Figure 1B‐a,b). In contrast, Mel‐KNiF_3_‐450‐500 and Mel‐KNiF_3_‐450‐550 exhibit an adsorption uptake in the high relative pressure range (*P*/*P*_0_ = 0.8–0.95) and clear hysteresis loops over *P*/*P*_0_ = 0.5–1.0, indicating the presence of cage‐type bottleneck mesoporous structures. The BJH pore size distributions show a dominant pore diameter of approximately 20 nm, closely matching the particle size of the KNiF_3_ templates (Figure 1C‐a,b). These results demonstrate that the pore diameter directly reflects the particle size of the K*M*F_3_ templates.

The Brunauer‐Emmett‐Teller (BET) surface areas of Mel‐KCoF_3_‐450‐500 and Mel‐KCoF_3_‐450‐550 are 54 and 60 m^2^ g^−1^, respectively, which are higher than those of bulk non‐porous C_3_N_4_ prepared at 500°C or 550°C without templates (5 or 10 m^2^ g^−1^, respectively; SEM images and N_2_ adsorption–desorption isotherms of these samples are shown in Figures S5 and S6). These results confirm the effective introduction of porosity by the KCoF_3_ templates. The Mel‐KNiF_3_‐450‐500 and Mel‐KNiF_3_‐450‐550 samples exhibit significantly higher surface areas of 136 and 105 m^2^ g^−1^, respectively. These values exceed those of both bulk C_3_N_4_ and the KCoF_3_‐templated samples, indicating that the smaller template particle size and the resulting mesoporous structure contribute to the enhanced surface area.

STEM images of the intermediate composites prior to template removal show that K*M*F_3_ particles are embedded within a polymer network without apparent porosity (Figures S7 and S8), suggesting that polymerization of melamine proceeds within the interparticle spaces of K*M*F_3_, resulting in the formation of a polymer framework that coats the template surfaces. This structural feature is essential for preserving the template‐defined structure during the thermal treatment. After complete removal of the K*M*F_3_ templates, the polymer framework preserves the template‐defined voids, leading to well‐defined porous structures. These observations demonstrate that K*M*F_3_ templates can be effectively applied in polymerization processes under high‐temperature conditions, enabling the generation of meso‐ or macroporous structures.

To clarify the role of the heating protocol, the mixture of melamine and K*M*F_3_ was directly heated at different temperatures (450°C, 500°C, and 550°C). For Mel‐KCoF_3_‐450 and Mel‐KNiF_3_‐450, although this temperature is insufficient for complete formation of C_3_N_4_ (Figure S9) [24, 25], these samples show porous structures consistent with the size of K*M*F_3_ and high surface areas (81 and 101 m^2^ g^−1^, respectively) (Figures S10 and S11), indicating that effective templating occurs at this stage. At 500°C, Mel‐KCoF_3_‐500 and Mel‐KNiF_3_‐500 yield porous structures with surface areas of 43 and 88 m^2^ g^−1^, respectively (Figures S12 and S13). In contrast, at 550°C, porous structures are not observed in Mel‐KCoF_3_‐550, whereas Mel‐KNiF_3_‐550 shows pore structures exceeding the size of the original KNiF_3_ templates (Figures S14 and S15).

This loss of porosity with increasing temperature is likely attributable to aggregation or sintering of the K*M*F_3_ particles, as seen in the SEM images (Figures S16 and S17), which reduces well‐defined interparticle spaces and prevents their effective replication as meso‐ or macropores. These findings suggest that polymerization proceeds at 450°C to form a C─N polymer network in the interparticle spaces and on the surface of K*M*F_3_, effectively suppressing aggregation/growth of K*M*F_3_ particles and stabilizing the C─N framework. Therefore, the two‐step strategy with low‐temperature polymerization plays a critical role in maintaining the template‐defined porous structure during subsequent thermal treatment.

**FIGURE 2:**
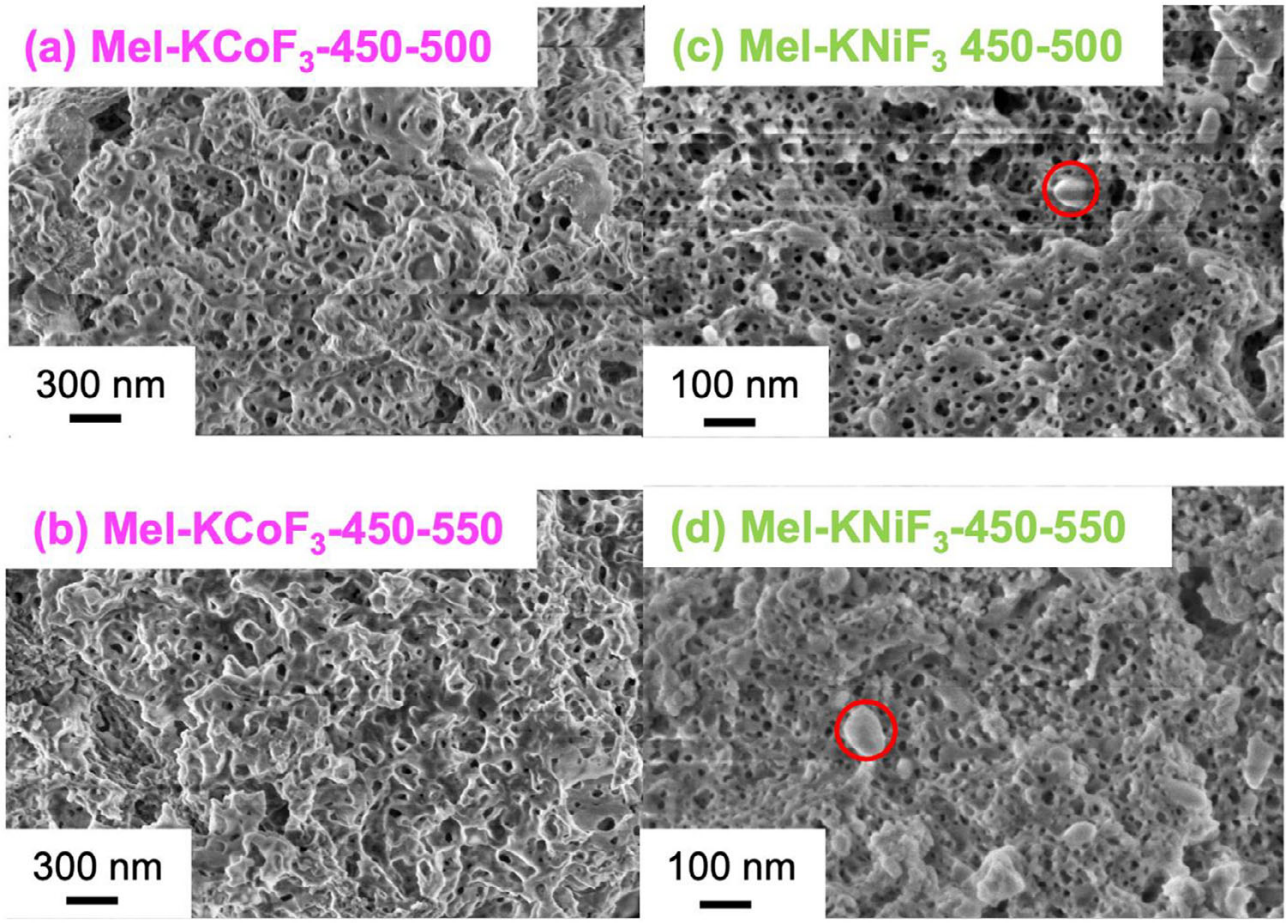
SEM images of (a) Mel‐KCoF_3_‐450‐500, (b) Mel‐KCoF_3_‐450‐550, (c) Mel‐KNiF_3_‐450‐500 and (d) Mel‐KNiF_3_‐450‐550.

Scanning electron microscopy (SEM) was conducted to examine the surface morphology of the obtained porous samples (Figure 2a–d). In all samples, open pores with sizes corresponding to those of the K*M*F_3_ template particles are observed. For the KNiF_3_‐templated samples, small particle‐like morphologies are also partially observed, as indicated by the red circles in Figure 2c,d. This observation in the KNiF_3_‐templated samples suggests that polymerization occurs not only within the interparticle spaces of the templates but also, to some extent, outside the templating regions, even in the presence of excess KNiF_3_.

**FIGURE 3:**
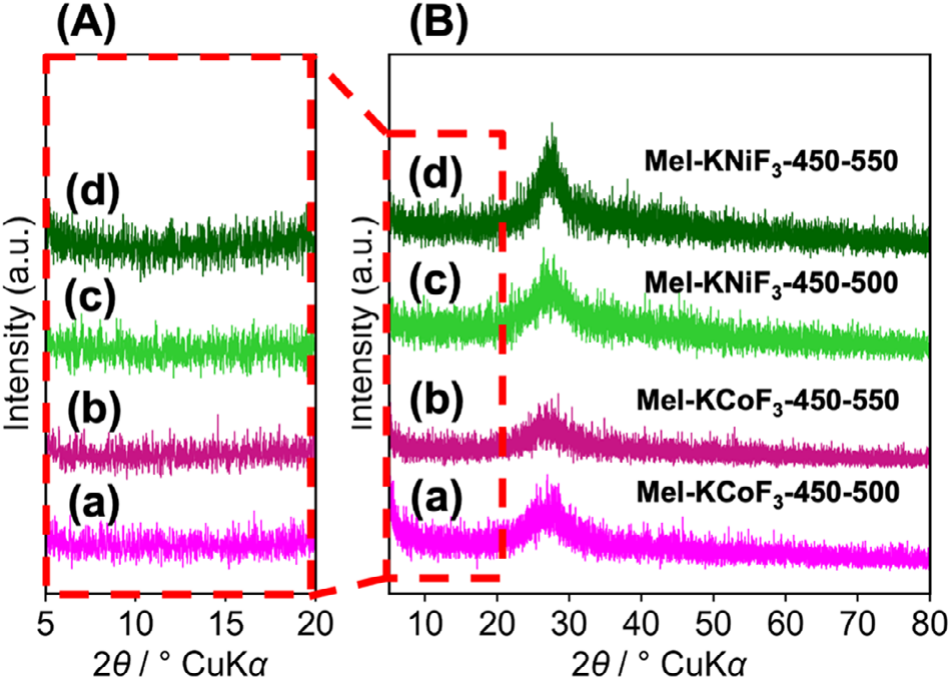
XRD patterns of (a) Mel‐KCoF_3_‐450‐500, (b) Mel‐KCoF_3_‐450‐550, (c) Mel‐KNiF_3_‐450‐500 and (d) Mel‐KNiF_3_‐450‐550. (A) measured at a scan rate of 2° min^−1^ over a 2*θ* range of 5°–30°, and (B) measured at a scan rate of 5° min^−1^ over a 2*θ* range of 5°–80°.

In this work, acid treatment with 6 m HCl was employed, although K*M*F_3_ templates can be easily removed by simple water treatment. This treatment is necessary for the removal of inorganic species, including metal carbodiimides, *M*NCN (*M* = Co, Ni), and metallic Co and Ni, which are formed via reactions with NH_3_ gas released from melamine during the thermal treatment, as evidenced by the XRD patterns of the intermediates after the polymerization (Figures S18 and S19) [26]. Treatment with 6 m HCl at room temperature leads to complete removal of crystalline K*M*F_3_ and major inorganic byproducts in the samples derived from KCoF_3_ and melamine (Figure 3a,b; the details are explained below). In contrast, the KNiF_3_‐templated intermediates require acid treatment at 60°C to ensure reliable removal of both the templates and byproducts. For Mel‐KNiF_3_‐450‐550 treated at room temperature, large particles are observed in the STEM images (Figure S20c,d), and the NiNCN phase is clearly detected by XRD (Figure S21). The presence of this phase results in a lower specific surface area (84 m^2^ g^−1^) (Figure S22) compared to the sample treated at 60°C (Figure 1B‐b). Similarly, for Mel‐KNiF_3_‐450‐500, the sample treated at room temperature exhibits a lower specific surface area (102 m^2^ g^−1^) (Figure S22) compared to that treated at 60°C (136 m^2^ g^−1^), although no obvious byproducts are observed in the STEM images (Figure S20a,b). This difference is likely due to the presence of trace amounts of residual byproducts. Therefore, acid treatment with 6 m HCl at 60°C is essential for the KNiF_3_‐derived samples.

To characterize the crystal structure, XRD measurements were conducted. The XRD patterns of bulk C_3_N_4_ prepared at 500°C and 550°C (Figure S23) show two characteristic peaks of g‐C_3_N_4_. Specifically, the diffraction peak at 2*θ* = 13° corresponds to the (100) plane of g‐C_3_N_4_, which arises from the in‐plane periodic arrangement of the heptazine structural units. The diffraction peak observed at 2*θ* = 27° is assigned to the (002) plane, originating from the interlayer stacking of the conjugated aromatic rings in g‐C_3_N_4_ [27, 28]. In contrast, the XRD patterns of the porous materials exhibit no diffraction peaks corresponding to the in‐plane periodic structure of g‐C_3_N_4_ and the crystalline K*M*F_3_ templates (Figure 3A), indicating that the K*M*F_3_ and other related byproducts are predominately removed by the acid treatment. Furthermore, the diffraction feature around 2*θ* = 27°–28° becomes markedly broadened and weakened, indicating a significant reduction in interlayer stacking order (Figure 3B). These observations suggest the formation of an amorphous carbon nitride framework, accompanied by a significant reduction in long‐range in‐plane ordering during the polymerization process in the presence of the K*M*F_3_ templates.

**FIGURE 4:**
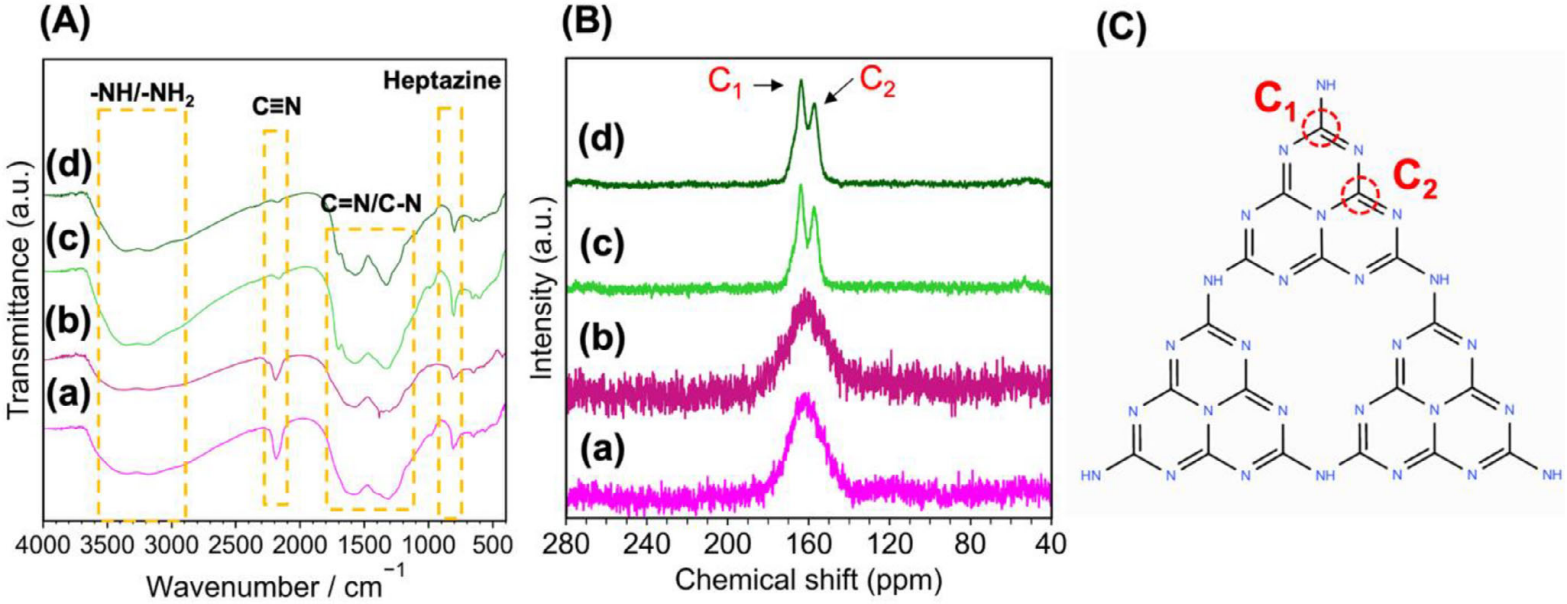
(A) FT‐IR spectra and (B) ^13^C CP/MAS NMR spectra of (a) Mel‐KCoF_3_‐450‐500, (b) Mel‐KCoF_3_‐450‐550, (c) Mel‐KNiF_3_‐450‐500 and (d) Mel‐KNiF_3_‐450‐550, and (C) Chemical structure of the heptazine units in g‐C_3_N_4_.

The chemical structures were investigated by FT‐IR spectroscopy. The spectra of C_3_N_4_‐500 and C_3_N_4_‐550 exhibit characteristic vibrational bands of g‐C_3_N_4_, including the out‐of‐plane bending mode of heptazine units at approximately 810 cm^−1^, the stretching modes of C─N heterocycles (C─N/C═N) in the range of 1200–1650 cm^−1^, and a broad absorption band between 3000 and 3400 cm^−1^ attributed to the stretching vibrations of terminal amino groups (Figure S24). In contrast, the templated samples retain these characteristic features, while they display noticeably weakened heptazine‐related bands and broadened C─N heterocycles (Figure 4A) [29, 30]. These observations suggest suppressed development of extended heptazine networks in the presence of K*M*F_3_ templates. In addition, absorption bands at approximately 2100 cm^−1^ assigned to cyanamide (─C≡N) terminal groups are observed for the Mel‐KCoF_3_ samples (Figure 4A‐a,b), further indicating incomplete condensation and the presence of terminal functionalities [31]. On the other hand, the cyanamide‐related band is much weaker in the Mel‐KNiF_3_ samples (Figure 4A‐c,d), which can be attributed to differences in acid‐washing conditions. More rigorous acid treatment at 60°C likely induces protonation of cyanamide terminal groups, reducing IR absorption band intensity. Indeed, Mel‐KNiF_3_‐450‐500 washed under milder conditions clearly exhibits this absorption band (Figure S25).

The structural disorder is further supported by solid‐state ^13^C CP/MAS NMR spectroscopy (Figure 4B). Bulk C_3_N_4_‐500 and C_3_N_4_‐550 exhibit well‐resolved C_1_ and C_2_ resonances at around 158 and 165 ppm (Figure S26). For the KCoF_3_‐templated samples (Figure 4B‐a,b), the resonances are completely broadened, suggesting a high degree of local structural heterogeneity. In contrast, for the KNiF_3_‐templated samples (Figure 4B‐c,d), discernible C_1_ and C_2_ features are observed and are more clearly resolved than those in the KCoF_3_‐templated samples. This difference in the local environment between the KCoF_3_‐ and KNiF_3_‐templated samples is likely related to the substantial variation in interparticle size between KNiF_3_ (10–30 nm) and KCoF_3_ (50–200 nm), which may lead to differences in the available reaction space during polycondensation and consequently influence the development of local structural order [32, 33, 34].

Even under milder acid‐washing conditions, Mel‐KNiF_3_‐450‐500 exhibits weak but discernible splitting of C_1_ and C_2_ resonances (Figure S27), suggesting that a certain degree of local structural order is present rather than a fully amorphous framework in the KNiF_3_‐templated samples. The NMR features of the KNiF_3_‐templated samples vary depending on the acid treatment conditions (room temperature or 60°C). The more rigorous treatment is accompanied by the disappearance of the cyanamide terminal groups in the FT–IR spectra, as described above (Figure S25), resulting in a more uniform local chemical environment and thereby making the local C_1_/C_2_ features more apparent.

Further investigation of the chemical structure of the samples was carried out by X‐ray photoelectron spectroscopy (XPS). First, wide survey spectra were recorded to identify the elemental composition of each sample (Figures S28–S30). All KCoF_3_‐ and KNiF_3_‐templated samples show signals corresponding to carbon and nitrogen, along with oxygen species likely introduced during acid washing and/or exposure to air. In addition, residual chlorine species are confirmed for the Mel‐KNiF_3_‐450‐500 and Mel‐KNiF_3_‐450‐550 samples, suggesting partial incorporation or surface adsorption of Cl originating from the HCl treatment.

The surface C/N atomic ratios are calculated from the relative sensitivity factor (RSF)‐corrected integrated peak areas of the C 1s and N 1s spectra in XPS. The resulting values are 0.72, 0.76, 0.74, and 0.74 for Mel‐KCoF_3_‐450‐500, Mel‐KCoF_3_‐450‐550, Mel‐KNiF_3_‐450‐500, and Mel‐KNiF_3_‐450‐550, respectively. For bulk C_3_N_4_ prepared at 500°C and 550°C, the C/N atomic ratios are 0.81 and 0.75, respectively. Although slightly higher ratios are observed for some samples, the values are overall close to the theoretical C/N ratio of g‐C_3_N_4_ (0.75). These results indicate that carbon nitride‐like C─N networks are formed under all conditions. The IR spectra confirm the formation of a heptazine framework, as described above, and suggest that the variations in the degree of structural fragmentation and/or distortion of the heptazine polymeric structure may account for the slight difference in the C/N ratio. In addition, the KCoF_3_‐ and KNiF_3_‐templated samples clearly contain metal species derived from the corresponding K*M*F_3_ templates (*M* = Co or Ni) as summarized in Table S1. The metal contents are quantified to be 1.00 and 1.05 at % for Mel‐KCoF_3_‐450‐500 and Mel‐KCoF_3_‐450‐550, respectively, and 1.35 and 2.09 at % for Mel‐KNiF_3_‐450‐500 and Mel‐KNiF_3_‐450‐550, respectively.

**FIGURE 5:**
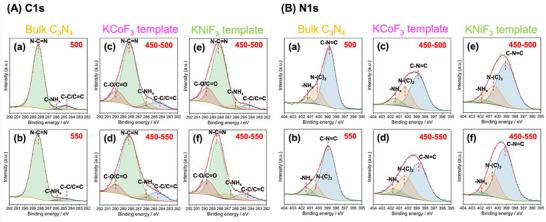
XPS spectra of (A) C 1s and (B) N 1s for (a) C_3_N_4_‐500, (b) C_3_N_4_‐550, (c) Mel‐KCoF_3_‐450‐500, (d) Mel‐KCoF_3_‐450‐550, (e) Mel‐KNiF_3_‐450‐500, and (f) Mel‐KNiF_3_‐450‐550.

Figure 5A,B shows the C 1s and N 1s XPS spectra of bulk C_3_N_4_, KCoF_3_‐templated samples, and KNiF_3_‐templated samples. For C 1s spectra, bulk C_3_N_4_ predominantly consists of three components, which are assigned to C─C/C═C, terminal C─NH*_x_* (*x* = 1, 2), and the N─C═N framework species (Figure 5A‐a,b). In contrast, the Mel‐KCoF_3_‐450‐500, Mel‐KCoF_3_‐450‐550, Mel‐KNiF_3_‐450‐500, and Mel‐KNiF_3_‐450‐550 samples exhibit an additional component, resulting in four distinct peaks at approximately 285, 286, 288, and 290 eV, corresponding to C─C, C─NH_x_, N─C═N, and oxidized terminal carbon species, respectively (Figure 5A‐c–f). Compared to bulk C_3_N_4_, the C 1s spectra of the K*M*F_3_‐templated samples show an increased contribution of C─NH_x_ (286 eV), indicating incomplete polycondensation of the polymer network. In addition, the appearance of C─O/C═O components (290 eV) suggests the formation of surface defects or partial oxidation, likely associated with acid treatment and exposure of edge sites.

In the N 1s spectrum (Figure 5B), bulk C_3_N_4_ can be deconvoluted into three components located at approximately 399, 400, and 402 eV, similar to those observed for the K*M*F_3_‐templated samples. These peaks are attributed to sp^2^‐hybridized nitrogen (C─N═C), tertiary nitrogen (N─(C)_3_) connecting the heptazine units, and amino nitrogen species (─NH_x_), respectively. In the K*M*F_3_‐templated samples, the relative intensity of the C─N═C component is clearly reduced compared to bulk C_3_N_4_. This suggests that although the fundamental heptazine units are formed in the presence of K*M*F_3_, the development of the extended conjugated network is suppressed, which is consistent with the formation of an amorphous framework in the K*M*F_3_‐templated samples.

The Co 2p XPS spectra of Mel‐KCoF_3_‐450‐500 and Mel‐KCoF_3_‐450‐550 (Figure S31a,b) exhibit two characteristic peaks assigned to Co 2p_3/2_ and Co 2p_1/2_ at approximately 782 and 797 eV, respectively, accompanied by their satellite peaks [35]. Similarly, the Ni 2p XPS spectra of Mel‐KNiF_3_‐450‐500 and Mel‐KNiF_3_‐450‐550 (Figure S31c,d) show Ni 2p_3/2_ and Ni 2p_1/2_ peaks centered at around 856 and 873 eV, together with the corresponding satellite features [36]. These spectral features indicate that Co and Ni are predominantly present in oxidized states. Previous studies have suggested that metal species in carbon nitride frameworks can coordinate with nitrogen‐containing sites [36, 37]. Although the present XPS data do not provide direct structural evidence, the observed oxidized states are consistent with metal species interacting with the nitrogen‐rich carbon nitride framework. Such interactions may interfere with the development of periodic stacking and in‐plane ordering of heptazine units.

Taken together with XRD, FT–IR, NMR, and XPS, these findings suggest that the K*M*F_3_‐templated samples possess a predominantly amorphous framework, likely arising from polymerization within the confined spaces between K*M*F_3_ particles, which restricts the development of long‐range heptazine ordering and results in incomplete polycondensation, as evidenced by the retention of terminal groups such as cyanamide. In addition, partial incorporation of metal species derived from K*M*F_3_ may additionally influence structural regularity.

The electrochemical glucose sensing performance of C_3_N_4_‐500, Mel‐KCoF_3_‐450‐500, and Mel‐KNiF_3_‐450‐500 was investigated to gain insight into how metal composition and pore structure influence glucose sensing behavior using a three‐electrode configuration. Cyclic voltammetry in blank 0.1 m KOH for C_3_N_4_‐500 reveals no distinct redox feature, whereas Mel‐KNiF_3_‐450‐500 and Mel‐KCoF_3_‐450‐500 exhibit characteristic redox peaks in the ranges of 0.4–0.6 V and 0.1–0.2 V, respectively, attributed to the oxidation/reduction of incorporated metal species (Figure S32) [38, 39, 40]. In contrast, upon addition of 5 mm glucose, both metal‐containing samples show higher anodic currents than those measured in the blank KOH solution over the measured potential range of 0.0–0.8 V (Figure S33), indicating metal‐mediated glucose oxidation under alkaline conditions [41].

**FIGURE 6:**
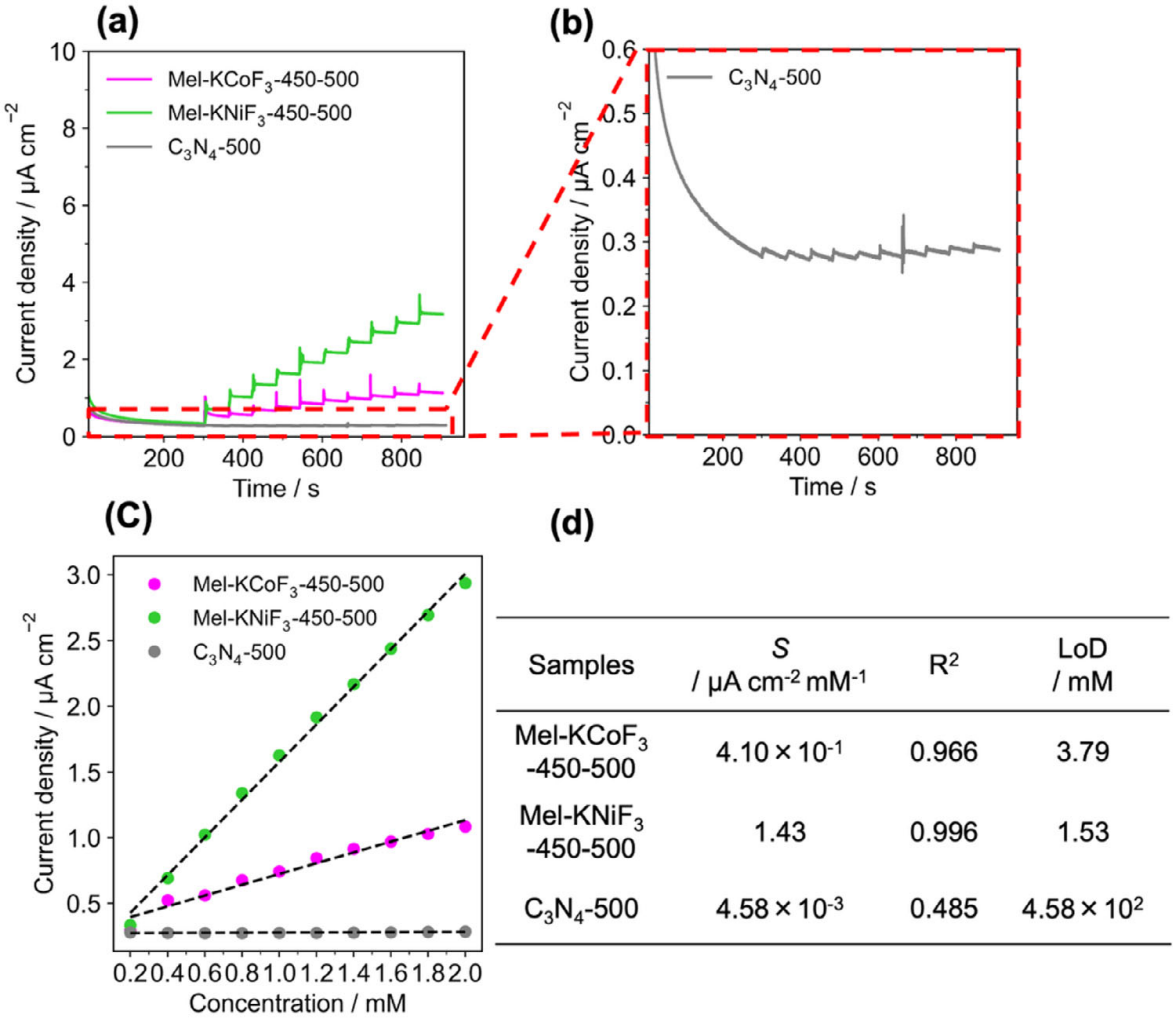
(a) Amperometric responses of Mel‐KCoF_3_‐450‐500, Mel‐KNiF_3_‐450‐500, and C_3_N_4_‐500 toward successive additions of glucose at 0.55 V (vs. Ag/AgCl) under stirring (900 rpm) in 0.1 m KOH, and (b) the enlarged view of C_3_N_4_‐500. (c) Corresponding calibration plots derived from the steady‐state currents. (d) Summary of sensitivity (*S*), coefficient of determination (R^2^), and limit of detection (LoD) for each sample.

Amperometric measurements were performed at 0.55 V (vs. Ag/AgCl) based on the CV results, where glucose oxidation is clearly distinguishable from the background current (Figure 6a,b). The measurements were conducted under stirring at 900 rpm to ensure sufficient mass transport, as lower stirring rates (e.g., 400 rpm) result in limited mass transport (Figure S34). Both Mel‐KNiF_3_‐450‐500 and Mel‐KCoF_3_‐450‐500 exhibit rapid and stable current increases upon successive glucose additions, reaching a steady state within a few seconds. The step current increased proportionally with glucose concentration, confirming concentration‐dependent oxidation behavior. In contrast, C_3_N_4_‐500 exhibits negligible current responses upon glucose addition, indicating its limited electrochemical activity toward glucose oxidation under the same conditions. Among the templated samples, Mel‐KNiF_3_‐450‐500 consistently displays higher current responses than Mel‐KCoF_3_‐450‐500 across the entire concentration range. Differential pulse voltammetry (DPV) measurements further support this concentration‐dependent response (Figure S35).

Calibration plots derived from the steady‐state currents show good linearity (Figure 6c). The sensitivities are 1.43 µA cm^−2^ mM^−1^ (R^2^ = 0.996) for Mel‐KNiF_3_‐450‐500 and 0.410 µA cm^−2^ mM^−1^ (R^2^ = 0.966) for Mel‐KCoF_3_‐450‐500. The limits of detection (LoD), calculated using LoD = 3σ/S, are 1.53 and 3.79 mM, respectively (Figure 6d). In contrast, C_3_N_4_‐500 exhibits an extremely low sensitivity (4.58 × 10^−3^ µA cm^−2^ mM^−1^) and poor linearity (R^2^ = 0.485), indicating negligible sensing capability under the same conditions. These results demonstrate that both templated samples have significantly better performance than pristine C_3_N_4_, confirming the effectiveness of the templating strategy in enhancing electrochemical sensing performance.

Mel‐KNiF_3_‐450‐500 exhibits higher sensitivity than Mel‐KCoF_3_‐450‐500. Transition metal species such as Ni and Co (e.g., Ni^2+^/Ni^3+^ and Co^2+^/Co^3+^) are generally known to facilitate glucose oxidation in alkaline media, as reported in previous studies [41, 42]. However, the observed difference between these two samples is not solely determined by the presence of such metal species. Rather, the superior performance of Mel‐KNiF_3_‐450‐500 is likely associated with its mesoporous structure, which results in higher specific surface area and enables more efficient exposure and utilization of electrochemically active sites. In contrast, the macroporous structure of Mel‐KCoF_3_‐450‐500 may limit the accessible surface area despite the presence of active species. Furthermore, the NMR results indicate differences in the local framework structure of C_3_N_4_ network between the two samples, which may influence charge transport through the C_3_N_4_ and interfacial charge‐transfer behavior during glucose oxidation. Although intrinsic differences in the activity of Ni and Co species and the NMR‐observed differences in the local C_3_N_4_ framework structure may also contribute, the sensing current is strongly governed by the number of electrochemically accessible reaction sites and the transport of glucose and OH^−^ to these sites. Therefore, the substantial difference in specific surface area arising from the porous structures strongly suggests that the pore‐structure‐dependent active‐site accessibility plays a dominant role in determining the sensing response.

## 3 Conclusions

In summary, we have developed a strategy for constructing amorphous carbon nitride with meso‐ or macroporous structures using K*M*F_3_ (*M* = Co or Ni) as templates. The pore sizes of the resulting materials are directly governed by the dimensions of the template particles, demonstrating precise structural replication. Importantly, we show that a two‐stage thermal treatment, incorporating an initial low‐temperature polymerization step, is essential for forming a C─N polymer framework within the interparticle spaces and along the surfaces of K*M*F_3_. This early‐stage polymerization stabilizes the template/precursor composites and preserves the template‐defined architecture during the subsequent high‐temperature treatment, which promotes further condensation into C_3_N_4_ without structural collapse under thermal conditions. The mesoporous KNiF_3_‐templated sample exhibits superior electrochemical glucose sensing performance, with higher sensitivity and a lower detection limit than the bulk and macroporous counterparts. This enhancement is attributed to its higher surface area and improved accessibility of electroactive Ni sites. These results suggest a clear relationship between pore architecture, metal‐site accessibility, and electrochemical response. Overall, this work provides new insight into the role of staged thermal treatment in high‐temperature polymerization processes and establishes a practical design principle for constructing porous carbon nitride with controlled pore structures and enhanced electrochemical functionality.

## 4 Experimental Section

### 4.1 Reagents

Cobalt(II) chloride hexahydrate (98%, CoCl_2_·6H_2_O, Fujifilm Wako Pure Chemical Co.), nickel(II) chloride hexahydrate (98%, NiCl_2_·6H_2_O, Fujifilm Wako Pure Chemical Co.), potassium fluoride dihydrate (98%, KF·2H_2_O, Sigma–Aldrich), ethylene glycol (99.5%, HO(CH_2_)_2_OH, Fujifilm Wako Pure Chemical Co.), ethanol (99.5%, C_2_H_5_OH, Fujifilm Wako Pure Chemical Co.), methanol (99.5%, CH_3_OH, Fujifilm Wako Pure Chemical Co.), melamine (99.0%, C_3_H_6_N_6_, Fujifilm Wako Pure Chemical Co.), hydrochloric acid (35–37 wt.%, HCl, Fujifilm Wako Pure Chemical Co.), D (+) glucose (98%, C_6_H_12_O_6_, Fujifilm Wako Pure Chemical Co.), 8 m potassium hydroxide solution (KOH, Fujifilm Wako Pure Chemical Co.), and Ketjenblack EC‐300J as conductive carbon black (Nanografi) were used as received without further purification.

### 4.2 Solvothermal Synthesis of Perovskite Fluorides

Based on a previously reported method [43], KF·2H_2_O (15 mmol) and MCl_2_·6H_2_O (6 mmol, *M* = Co or Ni) were added to ethylene glycol (40 mL) and sonicated at room temperature to obtain a homogeneously mixed solution. The resulting solution was transferred into a 50 mL Teflon‐lined stainless‐steel autoclave and subjected to solvothermal treatment. For the synthesis of KCoF_3_, the mixture was heated at 180°C for 20 h, whereas for KNiF_3_, the reaction was carried out at 120°C for 10 h. The resulting precipitates were collected, washed several times with ethanol and methanol, and dried under vacuum.

### 4.3 Preparation of Porous Carbon Nitride Using KMF_3_ as Templates

The as‐synthesized KCoF_3_ or KNiF_3_ was mixed with melamine in a weight ratio of 2:1. A small amount of ethanol was added dropwise to the mixture, which was then ground in a mortar until homogeneous. The resulting powder was placed in a tubular furnace and first heated at 450°C for 1 h, followed by further heating to 500°C or 550°C and maintained at these temperatures for 30 min. The calcination was carried out under an N_2_ atmosphere with a heating rate of 5°C min^−1^. After calcination, the resulting composites were treated with 6 m HCl to remove the K*M*F_3_ templates, followed by filtration and washing with deionized water and ethanol. Acid treatment was conducted at room temperature for 30 min for the KCoF_3_‐derived samples and at 60°C for 3 h for the KNiF_3_‐derived samples.

For comparison, the mixture of K*M*F_3_ (*M* = Co or Ni) and melamine was directly calcined at 500°C or 550°C for 2 h. After calcination, the resulting composites were treated with acid to remove the templates following the same procedure as described above, yielding the porous materials.

### 4.4 Preparation of Carbon Nitride

Bulk carbon nitride (C_3_N_4_) was synthesized by calcining melamine at 500°C or 550°C for 2 h under a nitrogen atmosphere with a heating rate of 5°C min^−1^.

### 4.5 Electrochemical Glucose Sensing

Electrochemical glucose sensing was carried out in 0.1 m KOH aqueous solution using a conventional three‐electrode configuration. A glassy carbon electrode (GCE, φ = 3 mm) served as the working electrode, a Pt wire as the counter electrode, and an Ag/AgCl (sat. KCl) electrode as the reference electrode. Catalyst inks were prepared by dispersing 4 mg of the catalyst and 1 mg of carbon as a conductive agent in a mixture containing 50 µL of 5 wt.% Nafion solution and 950 µL of water/2‐propanol (1:1, v/v), followed by ultrasonication for 30 min to obtain a homogeneous suspension. Four µL of the ink was drop‐cast onto the GCE surface and dried under ambient conditions. Amperometric measurements were conducted at a constant potential of 0.55 V (vs. Ag/AgCl) under continuous stirring at 900 rpm. Before glucose addition, the current was allowed to stabilize for 300 s. During the sensing experiments, 10 µL aliquots of a 1 m glucose stock solution were sequentially injected into 50 mL of electrolyte at 60 s intervals, corresponding to a concentration increment of 0.2 mm per addition. Differential pulse voltammetry (DPV) measurements were performed at glucose concentrations of 0, 1, 2, 5, and 10 mm to further confirm the concentration‐dependent response.

### 4.6 Characterizations

Transmission electron microscopy (TEM) images were obtained using a JEM 2100‐F microscope (JEOL) operating at 200 kV. Scanning electron microscopy (SEM) and scanning transmission electron microscopy (STEM) images were obtained using a Zeiss Gemini SEM 560 microscope. Nitrogen adsorption–desorption isotherms were measured at 77 K using a MicrotracBEL BELSORP Mini instrument. Prior to measurements, the samples were degassed under vacuum at 100°C for 24 h. Powder X‐ray diffraction (XRD) patterns were recorded on a Rigaku SmartLab diffractometer using Cu Kα radiation (λ = 0.15418 nm). Fourier‐transform infrared (FT‐IR) spectra were collected on a JASCO FT/IR‐4X spectrometer. Solid‐state ^13^C CP/MAS NMR spectra were acquired on a Bruker AVANCE NEO 500 spectrometer at 10 or 14 kHz. X‐ray photoelectron spectroscopy (XPS) measurements were carried out using a PHI Quantes (ULVAC‐PHI) instrument with monochromatic Al Kα radiation. Thermogravimetric (TG) analysis was performed using a TG‐DTA8122/H instrument (Rigaku) under an N_2_ atmosphere.

## Conflicts of Interest

The authors declare no conflicts of interest.

## Supporting information




**Supporting File**: smll74242‐sup‐0001‐SuppMat.docx.

## Data Availability

The data that support the findings of this study are available from the corresponding author upon reasonable request.
