# Supplementary material for: Design of Well‐Defined Meso‐ or Macroporous Carbon Nitride with an Amorphous Framework via Perovskite Fluoride Templating
Source: Small. 2026 Jun 19;22(45):e74242. doi: 10.1002/smll.74242 (PMC13472243; doi:10.1002/smll.74242)
Supplement: Supplementary file 1 [file SMLL-22-e74242-s001.docx]

**Supporting Information**

**Design of Well-Defined Meso- or Macroporous Carbon Nitride**

**with an Amorphous Framework *via* Perovskite Fluoride Templating**

Kazuya Kozumi*^a^*, Yusuke Asakura*^a^*^,^*, Yusuke Yamauchi*^a^*^,^*^b^*^,^*

*^a^Department of Materials Process Engineering, Graduate School of Engineering, Nagoya University, Furo-cho, Chikusa-ku, Nagoya, Aichi 464-8603, Japan*

*^b^Australian Institute for Bioengineering and Nanotechnology (AIBN), The University of Queensland, Brisbane, Queensland 4072, Australia*

E-mail: asa.y@nagoya-u.jp, y.yamauchi@uq.edu.au

**Contents**

**Figure S1**. STEM images of (a) KCoF_3_ and (b) KNiF_3_.

**Figure S2**. XRD patterns of KCoF_3_ and KNiF_3_.

**Figure S3**. TG curves of (a) KCoF_3_ and (b) KNiF_3_ measured up to 600 °C under a N_2_ atmosphere.

**Figure S4**. XRD patterns of KCoF_3_ and KNiF_3_ calcined at 600 °C.

**Figure S5**. SEM images of (a) C_3_N_4_-500 and (b) C_3_N_4_-550.

**Figure S6**. (a) N_2_ adsorption-desorption isotherms and (b) BJH pore size distributions of C_3_N_4_-500 and C_3_N_4_-550.

**Figure S7**. STEM images of intermediate products of (a) Mel-KCoF_3_-450-500 and (b) Mel-KCoF_3_-450-550.

**Figure S8**. STEM images of intermediate products of (a) Mel-KNiF_3_-450-500 and (b) Mel-KNiF_3_-450-550.

**Figure S9**. FT-IR spectra of (a) Mel-KCoF_3_-450 and (b) Mel-KNiF_3_-450.

**Figure S10**. STEM and SEM images of (a, c) Mel-KCoF_3_-450 and (b, d) Mel-KNiF_3_-450.

**Figure S11**. (a) N_2_ adsorption-desorption isotherms and (b) BJH pore size distributions of Mel-KCoF_3_-450 and Mel-KNiF_3_-450.

**Figure S12**. STEM images of (a) Mel-KCoF_3_-500 and (b) Mel-KNiF_3_-500.

**Figure S13**. (a) N_2_ adsorption-desorption isotherms and (b) BJH pore size distributions of Mel-KCoF_3_-500 and Mel-KNiF_3_-500.

**Figure S14**. STEM images of (a, b) Mel-KCoF_3_-550.

**Figure S15**. STEM images of (a, b) Mel-KNiF_3_-550.

**Figure S16**. SEM and STEM images of (a, b) KCoF_3_-500 and (c, d) KCoF_3_-550 obtained after TG measurements at the corresponding temperatures.

**Figure S17**. SEM and STEM images of (a, b) KNiF_3_-500 and (c, d) KNiF_3_-550 obtained after TG measurements at the corresponding temperatures.

**Figure S18**. XRD patterns of intermediate products of Mel-KCoF_3_-450-500 and Mel-KCoF_3_-450-550.

**Figure S19**. XRD patterns of intermediate products of Mel-KNiF_3_-450-500 and Mel-KNiF_3_-450-550.

**Figure S20**. STEM images of (a, b) Mel-KNiF_3_-450-500 and (c, d) Mel-KNiF_3_-450-550 washed with 6 M HCl at room temperature for 30 min.

**Figure S21**. XRD patterns of Mel-KNiF_3_-450-500 and Mel-KNiF_3_-450-550 washed with 6 M HCl at room temperature for 30 min.

**Figure S22**. (a) N_2_ adsorption-desorption isotherms and (b) BJH pore size distributions of Mel-KNiF_3_-450-500 and Mel-KNiF_3_-450-550 washed with 6 M HCl at room temperature for 30 min.

**Figure S23**. XRD patterns of C_3_N_4_-500 and C_3_N_4_-550.

**Figure S24**. FT-IR spectra of C_3_N_4_-500 and C_3_N_4_-550.

**Figure S25**. FT-IR spectra of Mel-KNiF_3_-450-500 washed with 6 M HCl at room temperature for 30 min.

**Figure S26**. ^13^C CP/MAS NMR spectra of C_3_N_4_-500 and C_3_N_4_-550.

**Figure S27**. ^13^C CP/MAS NMR spectrum of Mel-KNiF_3_-450-500 washed with 6 M HCl at room temperature for 30 min.

**Figure S28**. Wide survey XPS spectra of C_3_N_4_-500 and C_3_N_4_-550.

**Figure S29**. Wide survey XPS spectra of Mel-KCoF_3_-450-500 and Mel-KCoF_3_-450-550.

**Figure S30**. Wide survey XPS spectra of Mel-KNiF_3_-450-500 and Mel-KNiF_3_-450-550.

**Figure S31**. XPS spectra of Co 2p for (a) Mel-KCoF_3_-450-500 and (b) Mel-KCoF_3_-450-550, and Ni 2p for (c) Mel-KNiF_3_-450-500 and (d) Mel-KNiF_3_-450-550.

**Figure S32**. CV curves of C_3_N_4_-500, Mel-KCoF_3_-450-500 and Mel-KNiF_3_-450-500 in 0.1 M KOH solutions (blank) at a scan rate of 10 mV s^-1^.

**Figure S33**. CV curves of C_3_N_4_-500, Mel-KCoF_3_-450-500 and Mel-KNiF_3_-450-500 in 5 mM glucose solutions at a scan rate of 10 mV s^-1^.

**Figure S34**. Amperometric response of Mel-KCoF_3_-450-500 and Mel-KNiF_3_-450-500 at 400 rpm.

**Figure S35**. DPV measurements of (a) C_3_N_4_-500, (b) Mel-KCoF_3_-450-500 and (c) Mel-KNiF_3_-450-500.

**Figure S1**. STEM images of (a) KCoF_3_ and (b) KNiF_3_.

**Figure S2**. XRD patterns of KCoF_3_ and KNiF_3_.

**Figure S3**. TG curves of (a) KCoF_3_ and (b) KNiF_3_ measured up to 600 °C under a N_2_ atmosphere.

**Figure S4**. XRD patterns of KCoF_3_ and KNiF_3_ calcined at 600 °C.

**Figure S5**. SEM images of (a) C_3_N_4_-500 and (b) C_3_N_4_-550.

**Figure S6**. (a) N_2_ adsorption-desorption isotherms and

(b) BJH pore size distributions of C_3_N_4_-500 and C_3_N_4_-550.

**Figure S7**. STEM images of intermediate products of

(a) Mel-KCoF_3_-450-500 and (b) Mel-KCoF_3_-450-550.

**Figure S8**. STEM images of intermediate products of

(a) Mel-KNiF_3_-450-500 and (b) Mel-KNiF_3_-450-550.

**Figure S9**. FT-IR spectra of (a) Mel-KCoF_3_-450 and (b) Mel-KNiF_3_-450.

**Figure S10**. STEM and SEM images of (a, c) Mel-KCoF_3_-450 and (b, d) Mel-KNiF_3_-450.

**Figure S11**. (a) N_2_ adsorption-desorption isotherms and

(b) BJH pore size distributions of Mel-KCoF_3_-450 and Mel-KNiF_3_-450.

**Figure S12**. STEM images of (a) Mel-KCoF_3_-500 and (b) Mel-KNiF_3_-500.

**Figure S13**. (a) N_2_ adsorption-desorption isotherms and

(b) BJH pore size distributions of Mel-KCoF_3_-500 and Mel-KNiF_3_-500.

**Figure S14**. STEM images of (a, b) Mel-KCoF_3_-550.

**Figure S15**. STEM images of (a, b) Mel-KNiF_3_-550.

**Figure S16**. SEM and STEM images of (a, b) KCoF_3_-500 and (c, d) KCoF_3_-550

obtained after TG measurements at the corresponding temperatures.

**Figure S17**. SEM and STEM images of (a, b) KNiF_3_-500 and (c, d) KNiF_3_-550

obtained after TG measurements at the corresponding temperatures.

**Figure S18**. XRD patterns of intermediate products of

Mel-KCoF_3_-450-500 and Mel-KCoF_3_-450-550.

**Figure S19**. XRD patterns of intermediate products of

Mel-KNiF_3_-450-500 and Mel-KNiF_3_-450-550.

**Figure S20**. STEM images of (a, b) Mel-KNiF_3_-450-500 and (c, d) Mel-KNiF_3_-450-550

washed with 6 M HCl at room temperature for 30 min.

**Figure S21**. XRD patterns of Mel-KNiF_3_-450-500 and Mel-KNiF_3_-450-550

washed with 6 M HCl at room temperature for 30 min.

**Figure S22**. (a) N_2_ adsorption-desorption isotherms and (b) BJH pore size distributions of

Mel-KNiF_3_-450-500 and Mel-KNiF_3_-450-550 washed with 6 M HCl at room temperature for 30 min.

**Figure S23**. XRD patterns of C_3_N_4_-500 and C_3_N_4_-550.

**Figure S24**. FT-IR spectra of C_3_N_4_-500 and C_3_N_4_-550.

**Figure S25**. FT-IR spectra of Mel-KNiF_3_-450-500 washed with 6 M HCl

at room temperature for 30 min.

**Figure S26**. ^13^C CP/MAS NMR spectra of C_3_N_4_-500 and C_3_N_4_-550.

**Figure S27**. ^13^C CP/MAS NMR spectrum of Mel-KNiF_3_-450-500 washed with 6 M HCl

at room temperature for 30 min.

**Figure S28**. Wide survey XPS spectra of (a) C_3_N_4_-500 and (b) C_3_N_4_-550.

**Figure S29**. Wide survey XPS spectra of (a) Mel-KCoF_3_-450-500 and (b) Mel-KCoF_3_-450-550.

**Figure S30**. Wide survey XPS spectra of (a) Mel-KNiF_3_-450-500 and (b) Mel-KNiF_3_-450-550.

| Table S1. Elemental composition and atomic ratios | | | | |
| --- | --- | --- | --- | --- |
| Sample name | C / at% | N / at% | C/N ratio | M (= Co or Ni) / at% |
| Mel-KCoF_3_-450-500 | 39.3 | 54.5 | 0.72 | 1.00 |
| Mel-KCoF_3_-450-550 | 41.1 | 54.0 | 0.76 | 1.05 |
| Mel-KNiF_3_-450-500 | 38.8 | 52.6 | 0.74 | 1.35 |
| Mel-KNiF_3_-450-550 | 38.6 | 52.0 | 0.74 | 2.09 |
| C_3_N_4_-500 | 44.7 | 55.1 | 0.81 | - |
| C_3_N_4_-550 | 42.9 | 57.1 | 0.75 | - |

**Figure S31.** XPS spectra of Co 2p for (a) Mel-KCoF_3_-450-500 and (b) Mel-KCoF_3_-450-550,

and Ni 2p for (c) Mel-KNiF_3_-450-500 and (d) Mel-KNiF_3_-450-550.

**Figure S32**. CV curves of C_3_N_4_-500, Mel-KCoF_3_-450-500 and

Mel-KNiF_3_-450-500 in 0.1 M KOH solutions (blank) at a scan rate of 10 mV s^-1^.

**Figure S33**. CV curves of C_3_N_4_-500, Mel-KCoF_3_-450-500 and

Mel-KNiF_3_-450-500 in 5 mM glucose solutions at a scan rate of 10 mV s^-1^.

**Figure S34.** Amperometric response of Mel-KCoF_3_-450-500 and Mel-KNiF_3_-450-500 at 400 rpm.

**Figure S35.** DPV measurements of (a) C_3_N_4_-500, (b) Mel-KCoF_3_-450-500

and (c) Mel-KNiF_3_-450-500.
